# Audiometric Monitoring and Its Determinants in Patients Undergoing Chemoradiotherapy for Head and Neck Cancer

**DOI:** 10.3390/medsci14050593

**Published:** 2026-09-20

**Authors:** Anda-Ioana Morgovan, Nicolae Constantin Balica, Cristina Mihaela Negru, Kristine Guran, Alexandru Orasan, Mihaela Andreea Banta, Crina Oana Pintea, Mihaela Iuliana Ciortan (Sirbu), Horatiu Eugen Stefanescu

**Affiliations:** 1 Department of ENT, “Victor Babes” University of Medicine and Pharmacy Timisoara, 300041 Timisoara, Romania; anda.morgovan@umft.ro; 2 Department of Otolaryngology, “Victor Babes” University of Medicine and Pharmacy Timisoara, 300041 Timisoara, Romania; balica@umft.ro (N.C.B.); guran.kristine@umft.ro (K.G.); alexandru.orasan@umft.ro (A.O.); andreea.banta@umft.ro (M.A.B.); crina.pintea@umft.ro (C.O.P.); mihaela.sirbu@umft.ro (M.I.C.); stefanescu@umft.ro (H.E.S.)

**Keywords:** head and neck neoplasms, audiometry, healthcare disparities, aged, chemoradiotherapy

## Abstract

Background and Objectives: Audiometric monitoring is recommended for patients receiving ototoxic (chemo)radiotherapy for head and neck cancer (HNC), yet real-world uptake is poorly characterized and potentially inequitable. We quantified the uptake and timing of audiometric testing and examined rural–urban residence and age as candidate determinants. Materials and Methods: We conducted a single-center retrospective cohort study of 70 consecutive patients with HNC treated with radiotherapy between November 2024 and September 2025, comparing rural (*n* = 32) and urban (*n* = 38) residents. Outcomes were audiometric testing at treatment initiation, repeat audiometry during follow-up, and post-treatment hearing change. Analyses included Fisher’s exact and Mann–Whitney U tests, Spearman correlations, Firth-penalized logistic regression, inverse probability of treatment weighting (IPTW), restricted cubic splines, and sensitivity analyses. Results: Audiometry at treatment initiation was performed in 31/70 patients (44.3%) and repeat audiometry in only 10/70 (14.3%); among tested patients with documented dates, just 19.4% underwent audiometry before the first radiotherapy fraction, so that a true pre-exposure baseline existed for only 6/70 patients (8.6%). Uptake at initiation was 53.7% with concurrent cisplatin, 14.3% with carboplatin, and 36.4% with radiotherapy alone (*p* = 0.114). Uptake did not differ by residence (rural 40.6% vs. urban 47.4%; odds ratio (OR) 0.76, 95% confidence interval (CI) 0.30–1.96, *p* = 0.634), with an IPTW-weighted risk difference of −9.9% (95% CI −30.9 to +11.8). Age was the dominant determinant: uptake fell from 90.5% below 65 years to 24.5% at ≥65 years (adjusted OR per decade 0.32, 95% CI 0.15–0.71, *p* = 0.005), with significant non-linearity (*p* = 0.015). Pre-existing hearing loss was associated with lower testing (OR 0.29, 95% CI 0.10–0.91, *p* = 0.032). Post-treatment hearing change occurred in 54.3% overall (rural 46.9% vs. urban 60.5%, *p* = 0.336), comprising a subjective hearing complaint in 51.4% and audiometric deterioration in six of the nine patients with paired audiograms. Conclusions: Audiometric monitoring was incomplete, rarely obtained before irradiation, and steeply age-patterned, whereas rural residence was not associated with lower uptake; structured, exposure-based referral pathways, triggered by planned cisplatin and clinically relevant cochlear dose and applied irrespective of age, are warranted.

## 1. Introduction

Head and neck cancer (HNC) accounts for more than 900,000 new diagnoses and over 450,000 deaths worldwide each year, ranking among the most common malignancies globally [1]. The disease is dominated by squamous cell carcinoma arising from the mucosa of the oral cavity, pharynx, and larynx, is strongly linked to tobacco, alcohol, and human papillomavirus exposure, and typically presents at a locally advanced stage in patients of advancing age with substantial comorbidity [2]. Because the anatomic region concentrates the organs of speech, swallowing, and hearing, the quality of survivorship after treatment depends critically on how systematically functional sequelae are anticipated, detected, and rehabilitated.

For locally advanced disease, radiotherapy with concurrent platinum-based chemotherapy is the non-surgical standard of care, providing an absolute survival benefit confirmed across 107 randomized trials in the updated Meta-Analysis of Chemotherapy in Head and Neck Cancer, although the magnitude of benefit declines with age [3]. Pivotal trials additionally established that the survival gain of concurrent cisplatin is purchased with a marked increase in acute and late toxicity relative to radiotherapy alone [4]. This efficacy–toxicity trade-off makes structured toxicity surveillance an integral component, rather than an optional supplement, of curative-intent treatment.

Among these toxicities, auditory injury occupies a special position because it is common, cumulative, and frequently irreversible. Systematic review evidence indicates that sensorineural hearing loss develops in roughly 17–88% of patients after (chemo)radiotherapy, depending on definitions and audiometric rigor [5]; radiation dose to the cochlea is an established determinant, with a recommended mean-dose constraint of ≤45 Gy [6]; and chemoradiation-induced hearing loss remains a major concern with direct consequences for communication and quality of life [7]. Crucially, hearing loss can only be graded, attributed, and rehabilitated if audiometric data exist—making the audiogram itself the gateway intervention.

Professional guidelines therefore recommend baseline audiometry before ototoxic therapy, scheduled monitoring during treatment, and post-treatment evaluation, yet implementation studies show that ototoxicity monitoring programs face substantial service gaps, workflow barriers, and inconsistent referral pathways even in well-resourced systems [8]. Physician-facing analyses similarly document that treating specialists recognize the value of monitoring but encounter practical obstacles in embedding audiology within oncologic care [9]. Real-world data quantifying who actually receives audiometric testing during head and neck (chemo)radiotherapy—and when—remain remarkably scarce.

Whenever uptake of a beneficial service is incomplete, the question of equity follows. Rural patients with cancer experience well-documented disadvantages in access, participation, and survival [10], and rural–urban survival gaps persist across high-income countries [11]. Older patients constitute a second vulnerable group: management of elderly patients with locoregionally confined HNC is complicated by comorbidity, frailty, and systematic underrepresentation in trials [12], and population-based analyses demonstrate age-patterned deviations from guideline-concordant care [13]. Whether these equity concerns extend to supportive-care processes such as audiometric monitoring is unknown.

The present study addresses this gap in a contemporary, consecutive, single-center cohort treated in 2024–2025. Its novelty is threefold: it treats the audiogram as a measurable process-of-care outcome rather than merely a data source; it examines rural–urban residence and age jointly as candidate determinants using both penalized multivariable regression and propensity-score weighting; and it characterizes not only whether but also when audiometry was obtained relative to the first radiotherapy fraction, a timing dimension essential for valid toxicity attribution and grading [14]. Our objectives were to quantify the uptake and timing of audiometric monitoring, to test whether rural residence is associated with lower uptake, and to identify the dominant determinants of testing.

## 2. Materials and Methods

### 2.1. Study Design and Population

We performed a retrospective, observational cohort study at the tertiary academic otolaryngology service affiliated with the “Victor Babes” University of Medicine and Pharmacy, Timisoara, Romania, which serves a mixed urban and rural catchment population in western Romania. Consecutive adult patients with histologically confirmed head and neck malignancies who initiated external-beam radiotherapy between November 2024 and September 2025 and who were referred for otolaryngologic evaluation were identified from the departmental treatment registry. The study was conducted in accordance with the Declaration of Helsinki and approved by the Scientific Research Ethics Committee of the ‘Victor Babeș’ University of Medicine and Pharmacy, Timișoara, Romania (Approval No. 30, 19 April 2024; revised 13 May 2026). Informed consent was obtained from all participants involved in the study.

Demographic, oncologic, treatment, and audiologic data were abstracted from electronic medical records, radiotherapy treatment plans, and audiology charts using a standardized extraction form. Residence was classified as rural or urban according to the administrative status of the patient’s declared locality. Dates of the first and last radiotherapy fractions and of every audiometric examination were recorded verbatim, enabling reconstruction of testing timing relative to treatment. All records were reviewed by two investigators; discrepancies were resolved by consensus, and the final analysis dataset comprised 70 patients.

### 2.2. Eligibility Criteria and Group Allocation

Patients were eligible if they were aged 18 years or older, had a primary malignant tumor of the head and neck region (including salivary gland and sinonasal primaries and cervical metastases of unknown primary), received at least one fraction of external-beam radiotherapy, and had documented residence and clinical hearing assessment before and after treatment. Patients treated exclusively with brachytherapy or systemic therapy and patients without retrievable residence information were excluded. No patient meeting the eligibility criteria during the study window was excluded for missing outcome documentation.

The exposure of primary interest was residence, dichotomized as rural (*n* = 32) versus urban (*n* = 38). Concurrent systemic therapy (weekly cisplatin 40 mg/m^2^ as the departmental standard, or carboplatin in cisplatin-ineligible patients), induction chemotherapy, and prior oncologic surgery were recorded as covariates rather than grouping variables. For descriptive stratification of monitoring and hearing outcomes, patients were additionally categorized into three mutually exclusive ototoxic-exposure groups: concurrent cisplatin (*n* = 41), concurrent carboplatin (*n* = 7), and radiotherapy without concurrent systemic therapy (*n* = 22). Radiotherapy was delivered with volumetric techniques using simultaneous integrated boost or sequential schedules; for simultaneous integrated boost prescriptions, the highest dose level was analyzed, and sequential boosts were summed, and curative intent was defined as a prescribed dose ≥50 Gy without documented palliative intent.

### 2.3. Variables, Outcomes, and Data Collection

The primary outcome was performance of audiometric testing at treatment initiation, defined as a documented pure-tone audiogram obtained in the interval surrounding the start of radiotherapy as part of pre-treatment or early on-treatment work-up. Because most initial audiograms were obtained after the first fraction, we distinguished a true pre-treatment baseline audiogram (obtained before the first radiotherapy fraction and before any platinum exposure) from an initial on-treatment audiogram (obtained after radiotherapy had started); only the former was regarded as an unexposed baseline. Secondary outcomes were repeat audiometry during follow-up, any audiometric testing at any time, completion of both examinations (enabling paired comparison), and clinically documented post-treatment hearing change, defined as a new or worsened subjective hearing complaint corroborated by the treating otolaryngologist and/or audiometric deterioration where testing was available. The two components of this composite were also analyzed separately: (i) subjective hearing change, defined as a new or worsened hearing complaint documented by the treating otolaryngologist at any post-treatment visit, and (ii) objective audiometric deterioration, assessed only in patients with paired audiograms and defined as a ≥10 dB increase in air-conduction threshold at two or more adjacent test frequencies, or ≥20 dB at any single frequency, in either ear relative to the initial audiogram. Repeat audiometry was not protocolized during the study period; for every repeat examination, we recorded whether it had been ordered because of a new or worsened hearing complaint or clinical suspicion of hearing loss (symptom-triggered) or as scheduled follow-up independent of symptoms. For every audiogram, we computed the delay in days between the first radiotherapy fraction and the examination, with negative values denoting testing before irradiation began.

Candidate determinants comprised residence, age (continuous and dichotomized at 65 years), pre-treatment hearing loss (any documented subjective or audiometric impairment before the first fraction; in patients with an initial audiogram, severity was graded from the better-ear pure-tone average at 0.5, 1, 2, and 4 kHz as normal [≤25 dB HL], mild [26–40 dB HL], moderate [41–60 dB HL], or severe [>60 dB HL]), tumor subsite grouped as oropharynx, larynx/hypopharynx, and other sites, clinical T4 category, nodal positivity, concurrent chemoradiotherapy, concurrent cisplatin specifically, induction chemotherapy, prior oncologic surgery, prescribed dose, number of fractions, overall treatment time, and two cochlear dosimetric parameters extracted from the approved treatment plan: the maximum point dose (Dmax) and the mean dose (Dmean), each calculated for the left and right cochlea separately and then averaged across the two cochleae for analysis (bilateral average). Process outcomes potentially competing with monitoring—radiotherapy interruption, treatment abandonment, and death during observation—were also recorded.

### 2.4. Statistical Analysis

Continuous variables are summarized as medians with interquartile ranges (IQR) because Shapiro–Wilk testing and inspection of distributions indicated non-normality for age, dosimetric, and time variables; categorical variables are presented as counts and percentages. Comparisons between residence groups and between monitored and unmonitored patients used the Mann–Whitney U test for continuous variables, with the Hodges–Lehmann estimator as the effect size for location shift, and the Fisher exact test (or chi-square test for multicategory variables) for categorical variables; comparisons across the three ototoxic-exposure groups used the Fisher–Freeman–Halton exact test. Effect sizes for binary outcomes are expressed as odds ratios (OR) with 95% confidence intervals (CI) computed by the Woolf method with Haldane–Anscombe correction and as absolute risk differences with Wald 95% CI; monotonic associations were quantified with Spearman rank correlations.

Multivariable analysis of the primary outcome used Firth-penalized logistic regression to mitigate small-sample and separation bias, with residence, age (per decade), pre-treatment hearing loss, concurrent chemoradiotherapy, and oropharyngeal subsite as prespecified covariates. To address confounding of the residence contrast explicitly, we estimated a propensity score for rural residence from age, pre-treatment hearing loss, concurrent chemoradiotherapy, oropharyngeal subsite, and clinical T4 category, applied stabilized inverse probability of treatment weighting (IPTW), verified balance using standardized mean differences (SMD, target |SMD| < 0.10), and computed weighted outcome rates, risk differences, and odds ratios with robust bootstrap 95% CI. The shape of the age–uptake relationship was modeled with a restricted cubic spline with three degrees of freedom in a binomial generalized linear model, with likelihood-ratio tests for overall association and non-linearity.

Missing data were not imputed; analyses used all available cases with denominators reported throughout (cochlear dosimetry was unavailable for one patient and the number of concurrent cycles for two patients; residence, age, and all outcome variables were complete). Robustness of the residence contrast was addressed through prespecified sensitivity analyses restricting to curative-intent treatment, excluding deaths and treatment abandonment, and re-estimating the contrast for alternative monitoring definitions (any audiometry, repeat audiometry, completion of both examinations) and for the hearing-change outcome. All tests were two-sided with statistical significance set at *p* < 0.05; exact *p*-values are reported without multiplicity adjustment given the exploratory design, and computations were performed in Python 3.12 (Python Software Foundation, Wilmington, DE, USA) using the pandas 2.2.3, SciPy 1.14.1, statsmodels 0.14.4, and scikit-learn 1.5.2 libraries.

## 3. Results

### 3.1. Cohort Characteristics by Residence

The cohort included 32 rural (45.7%) and 38 urban (54.3%) patients (Table 1). Median age was 69.0 years in both groups (*p* = 0.800), and pre-treatment hearing loss was present in 71.9% and 76.3%, respectively (*p* = 0.786). Tumor-site distribution, T4 category, and nodal positivity were also similar between groups.

Treatment characteristics are summarized in Table 2. Prescribed dose, curative intent, overall treatment time, concurrent CRT, cisplatin use, induction chemotherapy, prior surgery, cochlear exposure, and RT interruption did not differ significantly by residence. Median cochlear Dmax (bilateral average) was 2.9 Gy in rural and 4.5 Gy in urban patients (*p* = 0.736), and median cochlear Dmean was 2.1 Gy and 3.0 Gy, respectively (*p* = 0.812); no patient exceeded the recommended mean cochlear dose constraint of 45 Gy (highest individual Dmean 38.6 Gy).

### 3.2. Audiometric Monitoring and Hearing Outcomes

Monitoring and hearing outcomes are shown in Table 3. Audiometry at treatment initiation was performed in 31/70 patients (44.3%), repeat audiometry in 10/70 (14.3%), any audiometry in 32/70 (45.7%), and both examinations in 9/70 (12.9%). A true pre-treatment baseline audiogram, obtained before the first radiotherapy fraction, was available in only 6/70 patients (8.6%; rural 2, urban 4), whereas the remaining 25 initial audiograms were obtained after radiotherapy had started and therefore do not represent an unexposed baseline. Of the 10 repeat audiograms, seven were symptom-triggered (ordered because of a new or worsened hearing complaint or clinical suspicion of hearing loss), and three were obtained as scheduled follow-up independent of symptoms, all three in patients receiving concurrent cisplatin. None of the monitoring measures differed significantly by residence. Hearing change occurred in 15/32 rural (46.9%) and 23/38 urban patients (60.5%; OR 0.58, 95% CI 0.23–1.49; *p* = 0.336). When the two components of this composite outcome were separated, a new or worsened subjective hearing complaint was documented in 36/70 patients (51.4%; rural 14/32, 43.8% vs. urban 22/38, 57.9%; *p* = 0.337), and objective audiometric deterioration was present in six of the nine patients with paired audiograms (rural 2/3, urban 4/6), in 4/6 symptom-triggered and 2/3 scheduled repeat examinations. Two patients, both treated with concurrent cisplatin, had audiometric deterioration without a subjective complaint.

**Figure 1:**
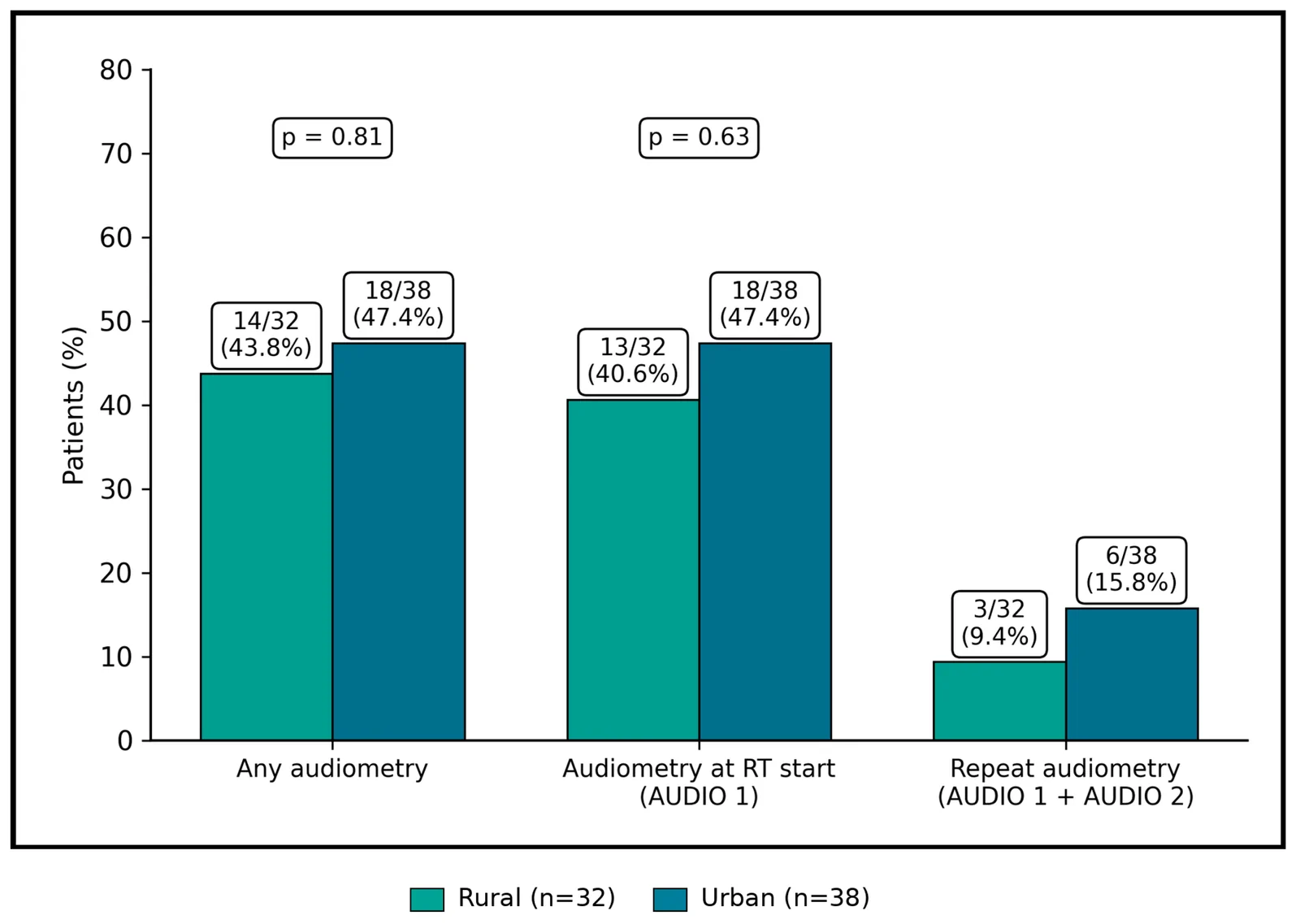
Audiometric monitoring cascade.

Figure 1 displays the monitoring cascade by residence. Any audiometry was obtained in 43.8% of rural and 47.4% of urban patients, audiometry at RT start in 40.6% and 47.4%, and both examinations in 9.4% and 15.8%, respectively; all within-step comparisons were non-significant.

**Figure 2:**
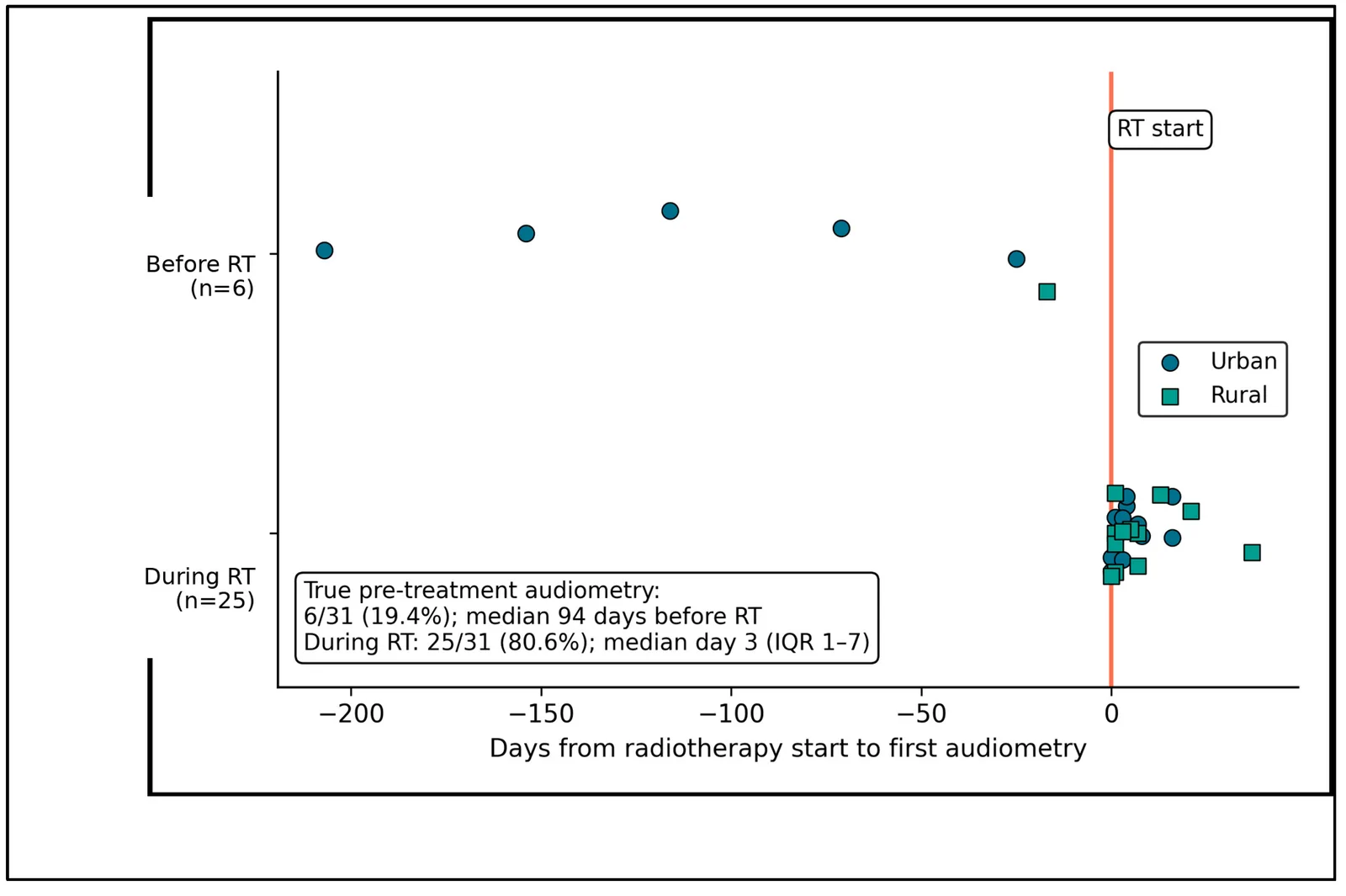
Timing of initial audiometry.

Timing of the initial audiogram is shown in Figure 2. Six of 31 tested patients (19.4%) underwent audiometry before RT, at a median of 94 days (range 12–158 days) before treatment, while 25/31 (80.6%) were tested after RT had started, at a median of day 3 (IQR 1–7; maximum 37 days). Four of the six pre-treatment audiograms had been obtained during the diagnostic work-up before induction chemotherapy, which accounts for the long interval and thus preceded any platinum exposure; three of the six were obtained within 90 days of the first fraction. Because no ototoxic therapy, otologic event, or new hearing complaint was documented between the examination and the start of RT in any of these patients, all six were accepted as pre-exposure baselines, although examinations older than 90 days would not satisfy the recommendation that a baseline be obtained shortly before the first ototoxic dose.

### 3.3. Determinants of Audiometric Monitoring

Univariable determinants are presented in Table 4. Tested patients were younger than untested patients (median 60.0 vs. 71.0 years, *p* < 0.001); uptake was 90.5% below age 65 and 24.5% at age ≥ 65. Pre-treatment hearing loss was associated with lower testing (OR 0.29, 95% CI 0.10–0.91; *p* = 0.032). This association was confounded by age: pre-treatment hearing loss was present in 9/21 patients (42.9%) younger than 65 years versus 43/49 (87.8%) aged ≥ 65 years (OR 0.10, 95% CI 0.03–0.35; *p* < 0.001), and within age strata testing did not differ by hearing status (age < 65 years: 8/9 [88.9%] with vs. 11/12 [91.7%] without hearing loss, *p* = 1.000; age ≥ 65 years: 11/43 [25.6%] vs. 1/6 [16.7%], *p* = 1.000). Among the 31 patients with an initial audiogram, the better-ear pure-tone average classified hearing as normal in 12 (38.7%), mildly impaired in nine (29.0%), moderately impaired in eight (25.8%), and severely impaired in two (6.5%); moderate or severe loss was present in 7/12 tested patients aged ≥ 65 years compared with 3/19 younger tested patients (*p* = 0.021). The severity of pre-treatment hearing loss could not be graded in the 39 untested patients, in whom hearing loss was documented clinically only. Concurrent cisplatin showed a non-significant positive association (OR 2.57, *p* = 0.087), while residence and other treatment variables were not significant.

Table 5 shows rural–urban comparisons within prespecified subgroups. No subgroup showed a significant residence association with audiometric testing, including strata defined by age, baseline hearing loss, CRT, the specific platinum agent (cisplatin or carboplatin), and tumor site.

Correlations are summarized in Table 6. Audiometry at treatment initiation was inversely correlated with age (ρ = −0.528, *p* < 0.001) but not with RT dose, overall treatment time, cochlear dose, or concurrent-cycle number. Hearing change correlated with cochlear Dmax (ρ = 0.244, *p* = 0.043); cochlear Dmean showed a similar, borderline correlation with hearing change (ρ = 0.229, *p* = 0.058) and no correlation with audiometry at initiation (ρ = −0.019, *p* = 0.878).

### 3.4. Multivariable and Propensity-Score Analyses

Firth regression results are shown in Table 7. In the adjusted model, age remained associated with lower testing (OR 0.32 per decade, 95% CI 0.15–0.71; *p* = 0.005). Rural residence (OR 0.64, *p* = 0.415), pre-treatment hearing loss (OR 1.39, *p* = 0.692), concurrent CRT (OR 1.07, *p* = 0.907), and oropharyngeal primary (OR 0.89, *p* = 0.846) were not significant.

IPTW results are presented in Table 8. Weighting reduced the maximum absolute SMD from 0.165 to 0.006. Weighted audiometry uptake was 38.4% in rural and 48.3% in urban patients (risk difference −9.9%, 95% CI −30.9 to +11.8; OR 0.67, 95% CI 0.26–1.64). Weighted hearing-change rates were 47.0% and 60.4%, respectively (OR 0.58, 95% CI 0.22–1.40).

**Figure 3:**
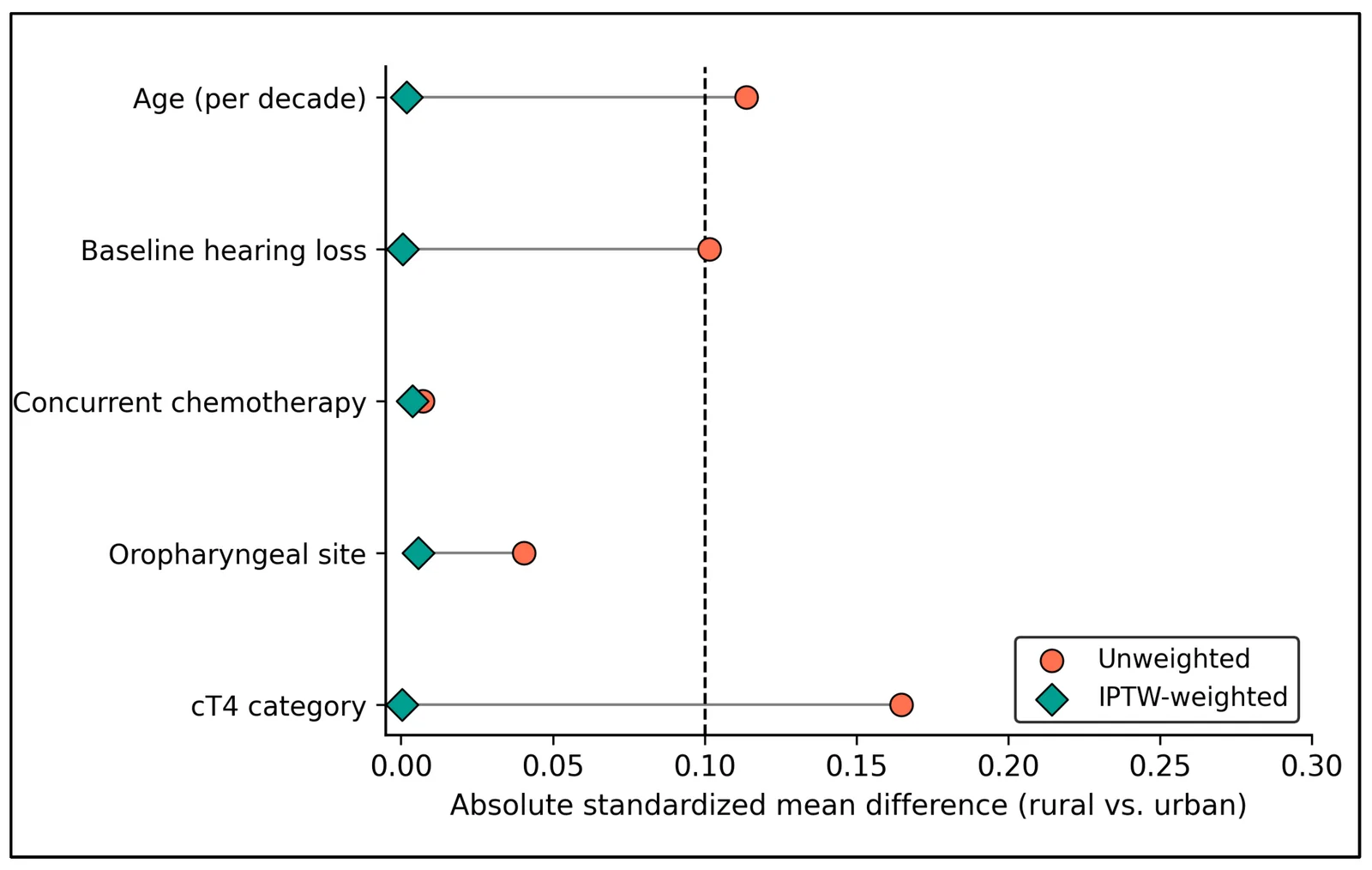
Covariate balance after IPTW. The dashed vertical line indicates the prespecified balance threshold of an absolute standardized mean difference of 0.10.

Figure 3 shows covariate balance before and after IPTW. The largest pre-weighting absolute SMD was 0.165, and all weighted SMDs were ≤0.006.

**Figure 4:**
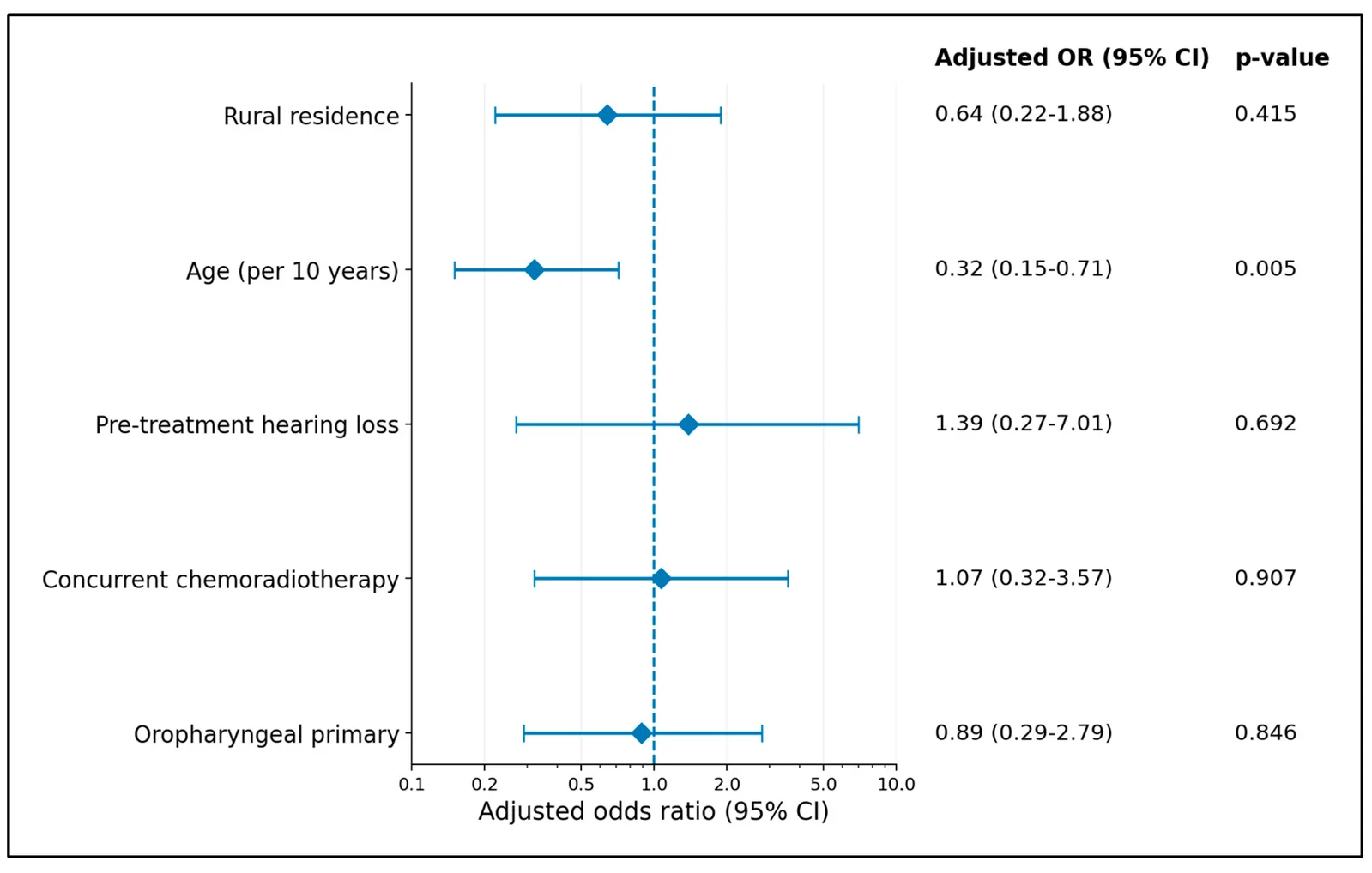
Adjusted determinants of audiometric monitoring. Diamonds and horizontal bars represent adjusted odds ratios with 95% confidence intervals from the Firth-penalized logistic regression model; the dashed vertical line indicates an odds ratio of 1 (no association).

Adjusted model estimates are displayed in Figure 4. Age was the only significant predictor of audiometry at treatment initiation (adjusted OR 0.32 per decade, 95% CI 0.15–0.71; *p* = 0.005); the remaining adjusted confidence intervals crossed 1. A separate restricted cubic spline analysis confirmed an overall association with age (*p* < 0.001) and significant non-linearity (*p* = 0.015).

### 3.5. Sensitivity Analyses

Sensitivity analyses are summarized in Table 9. Across alternative populations and monitoring definitions, rural-versus-urban ORs ranged from 0.55 to 0.86, and all *p*-values exceeded 0.33. The estimate for audiometry at initiation was 0.76 in the curative-intent subgroup and 0.79 after excluding deaths and treatment abandonment.

### 3.6. Monitoring and Hearing Outcomes by Ototoxic Exposure

Because cisplatin, carboplatin, and radiotherapy alone carry substantially different ototoxic risk, monitoring and hearing outcomes were also stratified by ototoxic-exposure group (Table 10). Audiometry at treatment initiation was obtained in 22/41 patients receiving concurrent cisplatin (53.7%), 1/7 receiving concurrent carboplatin (14.3%), and 8/22 treated with radiotherapy alone (36.4%; *p* = 0.114). A true pre-treatment baseline audiogram existed for only four cisplatin-treated patients (9.8%), no carboplatin-treated patients, and two patients treated with radiotherapy alone (9.1%), and repeat audiometry was obtained in 7/41 (17.1%), 0/7, and 3/22 (13.6%), respectively. Subjective hearing complaints were more frequent after cisplatin (24/41, 58.5%) than after carboplatin (3/7, 42.9%) or radiotherapy alone (9/22, 40.9%; *p* = 0.356), and audiometric deterioration was documented in five of six cisplatin-treated and one of three radiotherapy-alone patients with paired audiograms. Within the cisplatin group, uptake did not differ by residence (rural 10/21, 47.6% vs. urban 12/20, 60.0%; *p* = 0.536; Table 5).

## 4. Discussion

Two findings dominate this study: audiometric monitoring was incomplete across the cohort, and monitoring uptake did not differ materially between rural and urban residents once patients had reached the tertiary referral center. The latter result contrasts with the broader rural-oncology literature, which documents barriers related to travel, specialist availability, and fragmented services [15,16]. This apparent discrepancy is compatible with the design of our cohort, which begins after successful referral and treatment entry. Residence-related disadvantage may therefore operate upstream, while internal processes at the academic center may be more uniform. The overall monitoring deficit remains clinically important because survivorship care after head and neck cancer depends on identifying functional toxicities that can be treated or rehabilitated [17,18,19].

Advancing age was the strongest determinant of being left untested. Audiometry uptake fell from 90.5% in patients younger than 65 years to 24.5% among those aged 65 years or older, and age remained independently associated with lower testing after multivariable adjustment. The univariable association between pre-existing hearing loss and lower testing disappeared after age adjustment, suggesting that baseline hearing impairment largely tracked the same older population. The stratified analysis supports this interpretation: hearing loss was present in 88% of patients aged 65 years or older, and within age strata, patients with and without pre-treatment hearing loss were tested at similar rates, while moderate or severe baseline loss was common among the older patients who were tested. Rather than prompting audiometry, a pre-existing or age-related hearing deficit thus appears to have been regarded as an expected finding that did not warrant documentation, an attitude difficult to reconcile with the fact that patients with reduced cochlear reserve are precisely those in whom additional ototoxic injury has the greatest functional impact. This pattern runs counter to risk-based surveillance: objective ototoxicity studies, grading systems, and consensus frameworks depend on serial threshold measurements [20,21,22,23], and recommendations for audiologic surveillance do not exempt older adults [24]. Geriatric-oncology guidance similarly emphasizes more structured assessment in older patients rather than less [25,26].

The timing analysis identifies a second process problem that simple “tested versus untested” measures would miss. Only 19.4% of tested patients had an audiogram before the first radiotherapy fraction, whereas most initial examinations occurred after treatment had already started. Although the median on-treatment delay was only three days, these measurements are no longer pristine pre-exposure baselines, particularly for patients receiving cisplatin from the first treatment week. Only six patients (8.6% of the cohort) had a true pre-exposure baseline, and even these examinations were obtained a median of 94 days before radiotherapy, in most cases during the work-up before induction chemotherapy; although we accepted them as baselines because no ototoxic exposure intervened, an interval of this length falls short of current recommendations, which favor baseline testing shortly before the first ototoxic dose [8,24]. This limits the ability to attribute subsequent threshold changes to therapy and reduces comparability with formal ototoxicity grading systems [21,22,23,24]. A monitoring pathway should therefore specify not only that audiometry occur, but that baseline testing be completed before the first ototoxic exposure and followed by scheduled reassessment.

Stratifying by ototoxic exposure revealed a partially risk-concordant pattern: monitoring was most frequent in patients receiving concurrent cisplatin, the exposure with the highest ototoxic potential [5,7,20], and least frequent in the small carboplatin group. Even so, almost half of the cisplatin-treated patients had no audiogram at all, fewer than one in ten had a pre-exposure baseline, and fewer than one in five were re-tested, so that the group at greatest risk was also the group in which the largest absolute number of unmonitored patients accumulated. Because repeat audiometry was predominantly symptom-triggered rather than scheduled, the audiometric deterioration observed in most re-tested patients cannot be interpreted as an incidence estimate, and asymptomatic high-frequency loss in untested patients will have gone undetected.

The value of audiometric testing in this setting deserves emphasis, because it extends well beyond documentation. First, cisplatin- and radiation-induced hearing loss typically begins at high frequencies and is initially asymptomatic, so that a subjective complaint is a late and insensitive marker; systematic reviews report sensorineural loss in up to 88% of patients when audiometry is applied systematically [5,7]. Second, baseline and serial thresholds are the only means of applying ototoxicity grading scales and of attributing new loss to treatment rather than to presbycusis [20,21,22,23]. Third, during cisplatin-based treatment, detection of early threshold shifts can inform decisions on dose modification, substitution of the platinum agent, or otoprotective strategies before the loss reaches the speech frequencies [24]. Fourth, after treatment, audiometry is the gateway to timely amplification and auditory rehabilitation, which in older adults may attenuate the downstream consequences of hearing loss for communication, social participation, and cognition [27,28]. Finally, pure-tone audiometry is inexpensive, non-invasive, and widely available, so that its omission reflects a process failure rather than a resource constraint. These arguments apply irrespective of age and, in older patients with pre-existing loss, apply with greater rather than lesser force.

The propensity-score analysis strengthens the narrower conclusion that residence itself was not an important determinant of monitoring within this referred cohort. Baseline covariate imbalance was small before weighting and negligible afterward, and weighted estimates closely matched the unadjusted and regression-adjusted results. This convergence is reassuring for internal validity, but it should not be read as evidence that rural patients experience equal access across the full cancer-care pathway. Administrative rural–urban classification does not capture travel time, transportation, income, caregiver support, or the probability of reaching a tertiary center in the first place. Future multicenter studies should therefore distinguish pre-referral access from within-center delivery of supportive care.

The clinical implications are especially relevant for older adults, in whom untreated hearing impairment can worsen communication, social isolation, and cognitive vulnerability [27,28]. A practical response is an automatic, age-blind audiology referral that is triggered by the planned ototoxic exposure rather than by the radiotherapy prescription alone: any planned platinum-based chemotherapy (concurrent or induction) and any treatment plan in which the cochlea is expected to receive a clinically relevant dose, for example a mean cochlear dose approaching the recommended constraint of 45 Gy or a target volume abutting the temporal bone [6], should generate a referral, with baseline testing completed before the first ototoxic dose and a predefined follow-up interval. Patients treated with radiotherapy alone and low cochlear dose, who formed a substantial fraction of our cohort, could reasonably be managed with a single baseline audiogram and symptom-driven reassessment. The adjusted forest plot emphasizes that age, rather than residence or treatment-group variables, drove monitoring behavior in this dataset. Methodologically, the agreement between penalized regression, IPTW, subgroup analyses, and sensitivity analyses reduces the likelihood that the finding is an artifact of a single analytic choice [29,30]. These considerations should be viewed within the broader framework of functional outcomes and quality of life in head and neck cancer survivors. Multimodal treatment, combining surgery, radiotherapy, and systemic therapy, imposes a cumulative burden on speech, swallowing, and hearing, and contemporary outcome research increasingly evaluates function and patient-reported quality of life alongside oncologic control, as illustrated by recent patient-level analyses of functional outcomes after transoral robotic surgery for laryngeal cancer [31] and by survivorship frameworks for late effects [17,18]. Hearing is the least systematically measured of these functions, and unrecognized hearing loss compounds the communication difficulties already produced by treatment of the upper aerodigestive tract [27]. Audiometric surveillance therefore belongs alongside swallowing and voice assessment as a standard functional outcome measure in head and neck oncology. Prospective implementation studies are now needed to determine whether structured referral pathways improve both monitoring completion and timely rehabilitation.

## 5. Study Limitations

Several limitations must be acknowledged. This was a retrospective, single-center study of 70 patients, and although the event counts for the monitoring outcome were reasonably balanced, all effect estimates carry wide confidence intervals, and moderate residence effects—in either direction—cannot be excluded. Because the cohort comprises patients who successfully reached a tertiary referral center, upstream residence-based selection in diagnosis, referral, and treatment initiation is invisible to our design, and the null residence finding must not be generalized to the pre-referral pathway. The classification of residence relied on the administrative rural–urban status of the declared locality, which imperfectly captures travel distance, transportation access, and socioeconomic position, and no individual-level socioeconomic covariates were available. The hearing-change outcome integrated clinician-documented symptomatic assessment with audiometry where available and is therefore susceptible to ascertainment bias correlated with the monitoring outcome itself—a circularity we mitigated, but cannot eliminate, by analyzing monitoring and hearing outcomes separately. Although the subjective and audiometric components are now reported separately, objective deterioration could be assessed in only nine patients, most repeat audiograms were symptom-triggered, and the resulting proportions are descriptive only. Likewise, the severity of pre-treatment hearing loss could be graded only in tested patients, and for the 25 patients whose initial audiogram was obtained shortly after the first fraction, this grading approximates rather than establishes the pre-exposure status. The exposure-stratified analyses involve small subgroups, particularly the seven carboplatin-treated patients, and should be regarded as descriptive. Cochlear dosimetry was summarized as the bilateral average of Dmax and Dmean, which does not capture asymmetric exposure of the ipsilateral cochlea. Audiometry dates were occasionally affected by transcription errors requiring rule-based correction, and the timing analysis rests on the 31 tested patients. Finally, unmeasured confounding of the age effect by comorbidity, performance status, or cognitive impairment remains possible, and the findings await prospective, multicenter confirmation.

## 6. Conclusions

In this consecutive single-center cohort treated with modern (chemo)radiotherapy for head and neck cancer, audiometric monitoring was globally incomplete, rarely obtained before the first radiotherapy fraction, and almost never repeated, so that the paired measurements required for formal ototoxicity grading existed for only a small minority of patients; even among patients receiving concurrent cisplatin, fewer than one in ten had a true pre-exposure baseline. Rural residence was not associated with reduced monitoring or worse documented hearing outcomes among patients who reached the referral center, a null finding that proved stable across regression adjustment, propensity-score weighting, and multiple sensitivity analyses. Instead, advancing age emerged as the single dominant determinant of being left untested, following a threshold-like collapse around the conventional boundary of elderly status and inverting the risk logic that should govern surveillance. These findings argue for automatic, age-blind audiology referral anchored to the planned ototoxic exposure, namely concurrent or induction platinum chemotherapy and clinically relevant cochlear radiation dose, rather than to the radiotherapy prescription alone, with baseline testing completed before the first ototoxic dose, and for prospective evaluation of such structured pathways.

## Figures and Tables

**Table 1:** Baseline demographic and disease characteristics by residence.

Characteristic	Rural (*n* = 32)	Urban (*n* = 38)	Effect (95% CI)	*p*-Value
Age, years, median (IQR)	69.0 (58.8–73.5)	69.0 (67.0–72.8)	—	0.800
Age ≥ 65 years, *n* (%)	20 (62.5)	29 (76.3)	OR 0.52 (0.19–1.46)	0.296
Pre-treatment hearing loss, *n* (%)	23 (71.9)	29 (76.3)	OR 0.79 (0.27–2.33)	0.786
Tumor site, *n* (%)				0.986
Oropharynx	12 (37.5)	15 (39.5)		
Larynx/hypopharynx	13 (40.6)	15 (39.5)		
Other sites	7 (21.9)	8 (21.1)		
Clinical T4 category, *n* (%)	9 (28.1)	8 (21.1)	OR 1.47 (0.49–4.36)	0.580
Node-positive disease, *n* (%)	18 (56.2)	20 (52.6)	OR 1.16 (0.45–2.98)	0.813

IQR, interquartile range; OR, odds ratio; CI, confidence interval. Other sites include oral cavity, nasopharynx, salivary gland, paranasal sinus, and unknown primary. *p*-values are from the Mann–Whitney U test (continuous), Fisher exact test (binary), or chi-square test (multicategory).

**Table 2:** Treatment characteristics and cochlear exposure by residence.

Parameter	Rural (*n* = 32)	Urban (*n* = 38)	Effect (95% CI)	*p*-Value
Prescribed dose, Gy, median (IQR)	70.0 (69.5–70.0)	70.0 (70.0–70.0)	—	0.921
Curative-intent treatment, *n* (%)	29 (90.6)	36 (94.7)	OR 0.54 (0.08–3.45)	0.654
Overall treatment time, days, median (IQR)	49.0 (46.8–50.0)	50.0 (45.0–52.0)	HL −1.0	0.522
Concurrent chemoradiotherapy, *n* (%)	22 (68.8)	26 (68.4)	OR 1.02 (0.37–2.81)	1.000
Concurrent cisplatin, *n* (%)	21 (65.6)	20 (52.6)	OR 1.72 (0.65–4.55)	0.334
Concurrent cycles, median (IQR)	4.5 (2.2–5.8)	4.0 (0.0–5.0)	—	0.719
Induction chemotherapy, *n* (%)	7 (21.9)	12 (31.6)	OR 0.61 (0.21–1.79)	0.426
Prior oncologic surgery, *n* (%)	5 (15.6)	5 (13.2)	OR 1.22 (0.32–4.68)	1.000
Cochlear Dmax (bilateral average), Gy, median (IQR)	2.9 (1.9–15.9)	4.5 (2.0–12.1)	—	0.736
Cochlear Dmean (bilateral average), Gy, median (IQR)	2.1 (1.4–10.8)	3.0 (1.5–8.7)	—	0.812
Radiotherapy interruption, *n* (%)	2 (6.2)	4 (10.5)	OR 0.57 (0.10–3.32)	0.681

IQR, interquartile range; OR, odds ratio; CI, confidence interval; HL, Hodges–Lehmann shift estimate; Dmax, maximum point dose; Dmean, mean dose; both dosimetric parameters were extracted per cochlea from the treatment plan and averaged across the two cochleae. Cochlear dosimetry was available for 69 patients; concurrent cycles for 68 patients.

**Table 3:** Audiometric monitoring and hearing outcomes by residence.

Outcome	Rural (*n* = 32)	Urban (*n* = 38)	OR (95% CI)	*p*-Value
Audiometry at treatment initiation, *n* (%)	13 (40.6)	18 (47.4)	0.76 (0.30–1.96)	0.634
True pre-treatment baseline audiogram, *n* (%)	2 (6.2)	4 (10.5)	0.57 (0.10–3.32)	0.681
Initial audiogram after RT start, *n* (%)	11 (34.4)	14 (36.8)	0.90 (0.34–2.40)	1.000
Repeat audiometry during follow-up, *n* (%)	4 (12.5)	6 (15.8)	0.76 (0.19–2.99)	0.745
Any audiometry, *n* (%)	14 (43.8)	18 (47.4)	0.86 (0.34–2.20)	0.813
Both examinations completed, *n* (%)	3 (9.4)	6 (15.8)	0.55 (0.15–2.39)	0.494
Post-treatment hearing change, *n* (%)	15 (46.9)	23 (60.5)	0.58 (0.23–1.49)	0.336
Subjective hearing complaint, *n* (%)	14 (43.8)	22 (57.9)	0.57 (0.22–1.46)	0.337
Audiometric deterioration, *n*/*N* paired (%)	2/3 (66.7)	4/6 (66.7)	—	1.000
Death during observation, *n* (%)	1 (3.1)	1 (2.6)	—	1.000
Treatment abandonment, *n* (%)	0 (0.0)	1 (2.6)	—	1.000

OR, odds ratio; CI, confidence interval. Overall cohort rates: audiometry at treatment initiation 31/70 (44.3%); repeat audiometry 10/70 (14.3%); any audiometry 32/70 (45.7%); both examinations 9/70 (12.9%); true pre-treatment baseline audiogram 6/70 (8.6%); hearing change 38/70 (54.3%). RT, radiotherapy. Post-treatment hearing change is the composite of a subjective hearing complaint and/or audiometric deterioration; the two components are shown separately in the indented rows, with audiometric deterioration assessed only among the nine patients with paired audiograms.

**Table 4:** Univariable determinants of audiometry at treatment initiation.

Characteristic	Tested (*n* = 31)	Not Tested (*n* = 39)	Effect (95% CI)	*p*-Value
Age, years, median (IQR)	60.0 (53.5–68.5)	71.0 (69.0–76.0)	HL −10.0	<0.001
Age ≥ 65 years, *n* (%)	12 (38.7)	37 (94.9)	OR 0.03 (0.01–0.18)	<0.001
Pre-treatment hearing loss, *n* (%)	19 (61.3)	33 (84.6)	OR 0.29 (0.10–0.91)	0.032
Rural residence, *n* (%)	13 (41.9)	19 (48.7)	OR 0.76 (0.30–1.96)	0.634
Concurrent cisplatin, *n* (%)	22 (71.0)	19 (48.7)	OR 2.57 (0.93–6.63)	0.087
Concurrent chemoradiotherapy, *n* (%)	23 (74.2)	25 (64.1)	OR 1.61 (0.57–4.53)	0.442
Oropharyngeal primary, *n* (%)	10 (32.3)	17 (43.6)	OR 0.62 (0.23–1.66)	0.459
Clinical T4 category, *n* (%)	8 (25.8)	9 (23.1)	OR 1.16 (0.39–3.48)	1.000
Curative-intent treatment, *n* (%)	27 (87.1)	38 (97.4)	OR 0.18 (0.04–1.61)	0.163
Induction chemotherapy, *n* (%)	10 (32.3)	9 (23.1)	OR 1.59 (0.55–4.60)	0.428
Cochlear Dmax (bilateral average), Gy, median (IQR)	4.2 (2.1–13.4)	4.5 (1.8–15.4)	—	0.932
Cochlear Dmean (bilateral average), Gy, median (IQR)	2.7 (1.6–9.2)	2.9 (1.3–10.6)	—	0.884

IQR, interquartile range; OR, odds ratio; CI, confidence interval; HL, Hodges–Lehmann shift estimate; Dmax, maximum point dose; Dmean, mean dose (both averaged across the two cochleae). *p*-values from Mann–Whitney U test (continuous) or Fisher exact test (categorical). Cochlear dosimetry available for 69 patients.

**Table 5:** Rural versus urban audiometric testing at treatment initiation within prespecified subgroups.

Subgroup	*n*	Rural Tested, *n* (%)	Urban Tested, *n* (%)	OR (95% CI)	*p*-Value
All patients	70	13 (40.6)	18 (47.4)	0.76 (0.30–1.96)	0.634
Age < 65 years	21	10 (83.3)	9 (100.0)	0.00 (0.01–5.21) ^1^	0.486
Age ≥ 65 years	49	3 (15.0)	9 (31.0)	0.39 (0.11–1.72)	0.313
Pre-treatment hearing loss	52	7 (30.4)	12 (41.4)	0.62 (0.21–1.97)	0.563
No pre-treatment hearing loss	18	6 (66.7)	6 (66.7)	1.00 (0.16–6.28)	1.000
Concurrent chemoradiotherapy	48	10 (45.5)	13 (50.0)	0.83 (0.28–2.56)	0.780
Concurrent cisplatin	41	10 (47.6)	12 (60.0)	0.61 (0.18–2.09)	0.536
Concurrent carboplatin	7	0 (0.0)	1 (16.7)	1.22 (0.03–48.20) ^1^	1.000
Radiotherapy alone	22	3 (30.0)	5 (41.7)	0.60 (0.12–3.41)	0.675
Oropharyngeal primary	27	4 (33.3)	6 (40.0)	0.75 (0.17–3.53)	1.000
Non-oropharyngeal primary	43	9 (45.0)	12 (52.2)	0.75 (0.23–2.46)	0.763

OR, odds ratio; CI, confidence interval. *p*-values from Fisher exact tests within each stratum. ^1^ Zero-cell strata (all nine urban patients tested in the <65-year stratum; no rural carboplatin-treated patient tested); Haldane–Anscombe-corrected intervals are reported. The cisplatin and carboplatin strata are mutually exclusive subsets of the concurrent chemoradiotherapy stratum.

**Table 6:** Spearman rank correlation matrix among age, treatment variables, monitoring, and the hearing outcome.

Variable	Age	RT Dose	OTT	Cochlear Dmax	Concurrent Cycles	Audiometry at Initiation
RT dose	−0.047 (0.700)	—				
OTT	−0.140 (0.252)	0.672 (<0.001)	—			
Cochlear Dmax	−0.203 (0.095)	0.349 (0.003)	0.313 (0.009)	—		
Concurrent cycles	−0.179 (0.145)	0.381 (0.001)	0.270 (0.027)	0.275 (0.024)	—	
Audiometry at initiation	−0.528 (<0.001)	−0.082 (0.502)	0.063 (0.606)	−0.011 (0.929)	−0.004 (0.975)	—
Hearing change	0.098 (0.419)	0.103 (0.394)	0.011 (0.929)	0.244 (0.043)	0.176 (0.151)	−0.048 (0.694)

Values are Spearman ρ (*p*-value). RT, radiotherapy; OTT, overall treatment time; Dmax, maximum point dose (bilateral average). Pairwise complete observations (*n* = 68–70).

**Table 7:** Firth-penalized logistic regression for audiometry at treatment initiation.

Predictor	Unadjusted OR (95% CI)	*p*-Value	Adjusted OR (95% CI) ^1^	*p*-Value
Rural residence	0.77 (0.30–1.98)	0.584	0.64 (0.22–1.88)	0.415
Age, per 10 years	0.34 (0.18–0.63)	<0.001	0.32 (0.15–0.71)	0.005
Pre-treatment hearing loss	0.30 (0.10–0.93)	0.037	1.39 (0.27–7.01)	0.692
Concurrent chemoradiotherapy	1.57 (0.56–4.42)	0.391	1.07 (0.32–3.57)	0.907
Oropharyngeal primary	0.63 (0.24–1.68)	0.353	0.89 (0.29–2.79)	0.846
Clinical T4 category	1.16 (0.39–3.48)	0.789	—	—

OR, odds ratio; CI, confidence interval. ^1^ Adjusted model (*n* = 70) includes rural residence, age, pre-treatment hearing loss, concurrent chemoradiotherapy, and oropharyngeal primary.

**Table 8:** Inverse probability of treatment weighting analysis of residence contrasts.

Outcome	Rural, Weighted %	Urban, Weighted %	Risk Difference, % (95% CI)	OR (95% CI)
Audiometry at treatment initiation	38.4	48.3	−9.9 (−30.9 to +11.8)	0.67 (0.26–1.64)
Post-treatment hearing change	47.0	60.4	−13.3 (−35.4 to +8.2)	0.58 (0.22–1.40)

OR, odds ratio; CI, bootstrap 95% confidence interval. Stabilized weights from a propensity score for rural residence (age, pre-treatment hearing loss, concurrent chemoradiotherapy, oropharyngeal primary, clinical T4 category); propensity-score range 0.40–0.59; maximum absolute standardized mean difference 0.165 before and 0.006 after weighting.

**Table 9:** Sensitivity analyses for the association between rural residence and audiometric monitoring.

Analysis	*n*	Rural, *n*/*N* (%)	Urban, *n*/*N* (%)	OR (95% CI)	*p*-Value
Primary: audiometry at initiation	70	13/32 (40.6)	18/38 (47.4)	0.76 (0.30–1.96)	0.634
Curative-intent treatment only	65	11/29 (37.9)	16/36 (44.4)	0.76 (0.28–2.08)	0.623
Excluding deaths and abandonment	67	12/31 (38.7)	16/36 (44.4)	0.79 (0.30–2.08)	0.804
Outcome: any audiometry	70	14/32 (43.8)	18/38 (47.4)	0.86 (0.34–2.20)	0.813
Outcome: repeat audiometry	70	4/32 (12.5)	6/38 (15.8)	0.76 (0.19–2.99)	0.745
Outcome: both examinations	70	3/32 (9.4)	6/38 (15.8)	0.55 (0.15–2.39)	0.494
Outcome: hearing change	70	15/32 (46.9)	23/38 (60.5)	0.58 (0.23–1.49)	0.336

OR, odds ratio; CI, confidence interval. *p*-values from Fisher exact tests.

**Table 10:** Audiometric monitoring and hearing outcomes by ototoxic-exposure group.

Outcome	Cisplatin (*n* = 41)	Carboplatin (*n* = 7)	RT Alone (*n* = 22)	*p*-Value
Audiometry at treatment initiation, *n* (%)	22 (53.7)	1 (14.3)	8 (36.4)	0.114
True pre-treatment baseline audiogram, *n* (%)	4 (9.8)	0 (0.0)	2 (9.1)	1.000
Initial audiogram after RT start, *n* (%)	18 (43.9)	1 (14.3)	6 (27.3)	0.219
Repeat audiometry during follow-up, *n* (%)	7 (17.1)	0 (0.0)	3 (13.6)	0.689
Both examinations completed, *n* (%)	6 (14.6)	0 (0.0)	3 (13.6)	0.764
Post-treatment hearing change (composite), *n* (%)	26 (63.4)	3 (42.9)	9 (40.9)	0.179
Subjective hearing complaint, *n* (%)	24 (58.5)	3 (42.9)	9 (40.9)	0.356
Audiometric deterioration, *n*/*N* paired (%)	5/6 (83.3)	0/0 (—)	1/3 (33.3)	0.226 ^1^

RT, radiotherapy. Exposure groups are mutually exclusive; concurrent chemoradiotherapy comprised cisplatin (*n* = 41) or carboplatin (*n* = 7). *p*-values from Fisher–Freeman–Halton exact tests across the three groups. Audiometric deterioration is reported among patients with paired audiograms (*n* = 9). ^1^ Cisplatin versus radiotherapy alone (Fisher exact test); no carboplatin-treated patient had paired audiograms.

## Data Availability

The data presented in this study are available on reasonable request from the corresponding author. The data are not publicly available due to patient privacy restrictions.
